# Blood Pressure Modulation With Low-Intensity Focused Ultrasound Stimulation to the Vagus Nerve: A Pilot Animal Study

**DOI:** 10.3389/fnins.2020.586424

**Published:** 2020-11-12

**Authors:** Ning Ji, Wan-Hua Lin, Fei Chen, Lisheng Xu, Jianping Huang, Guanglin Li

**Affiliations:** ^1^CAS Key Laboratory of Human-Machine Intelligence-Synergy Systems and Research Center for Neural Engineering, Shenzhen Institutes of Advanced Technology (SIAT), Chinese Academy of Sciences (CAS), and the SIAT Branch, Shenzhen Institute of Artificial Intelligence and Robotics for Society, Shenzhen, China; ^2^College of Medicine and Biological Information Engineering, Northeastern University, Shenyang, China; ^3^Department of Electrical and Electronic Engineering, Southern University of Science and Technology, Shenzhen, China

**Keywords:** blood pressure management, low-intensity focused ultrasound stimulation, vagus nerve, neuromodulation, hypertension animal study

## Abstract

**Objective:**

For hypertensive individuals, their blood pressure (BP) is often managed by taking medications. However, antihypertensive drugs might cause adverse effects such as congestive heart failure and are ineffective in significant numbers of the hypertensive population. As an alternative method for hypertension management, non-drug devices-based neuromodulation approaches such as functional electrical stimulation (FES) have been proposed. The FES approach requires the implantation of a stimulator into the body. One recently emerging technique, called low-intensity focused ultrasound stimulation (FUS), has been proposed to non-invasively modulate neural activities. In this pilot study, the feasibility of adopting low-intensity FUS neuromodulation for BP regulation was investigated using animal models.

**Methods:**

A FUS system was developed for BP modulation in rabbits. For each rabbit, the low-intensity FUS with different acoustic intensities was used to stimulate its exposed left vagus nerve, and the BP waveform was synchronously recorded in its right common carotid artery. The effects of the different FUS intensities on systolic blood pressure (SBP), diastolic blood pressure (DBP), mean blood pressure (MAP), and heart rate (HR) were extensively examined from the BP recordings.

**Results:**

The results demonstrated that the proposed FUS method could successfully induce changes in SBP, DBP, MAP, and HR values. When increasing acoustic intensities, the values of SBP, DBP, and MAP would tend to decrease more substantially.

**Conclusion:**

The findings of this study suggested that BP could be modulated through the FUS, which might provide a new way for non-invasive and non-drug management of hypertension.

## Introduction

High blood pressure (BP) is one of the leading causes of morbidity and mortality worldwide. Clinically, a common way for BP management is to regularly take antihypertensive medications for the hypertensive population. While a variety of antihypertensive drugs could effectively regulate BP with a primary goal to prevent the occurrence of cardiovascular and cerebrovascular complications such as stroke, a long-term medication of antihypertensive administration may cause some potential side effects such as congestive heart failure, depression, immunological disease, orthostatic symptoms, palpitations, precipitate angina, sexual dysfunction, and syncope (Husserl and Franz, 1981; Marc et al., 2019). Furthermore, some of the existing antihypertensive drugs have been reported to be associated with an increased risk of myocardial infarction (Psaty et al., 1995) as well as ischemic stroke (Klungel et al., 2001). On the other hand, for a substantial portion of patients with hypertension, their BP is uncontrolled by currently available antihypertensive drugs, which are designated as having resistant hypertension (Bisognano et al., 2011).

Owing to those potential side effects of taking antihypertensive medications regularly for a long time (even during the rest of life) and the issue of resistant hypertension, device-based non-drug neuromodulation approaches have been proposed and developed for the administration of resistant hypertension. This is because resistant hypertension is mainly a neurogenic disease characterized by enhanced sympathetic nerve activity. Thus, novel neuromodulation approaches targeting sympathetic nerve inhibition might be potential for the treatment of resistant hypertension. Tremendous evidences have proved that neuromodulation techniques such as functional electrical stimulation (FES) of the carotid baroreceptor (Scheffers et al., 2010; Bisognano et al., 2011; Lohmeier and Iliescu, 2011; Bakris et al., 2012; Hoppe et al., 2012)/vagus nerve (Plachta et al., 2014; Gierthmuehlen et al., 2016; Annoni et al., 2019) and renal sympathetic denervation (RSD) by different devices and techniques [including surgical sympathectomy (Smithwick, 1948), laparoscopic sympathectomy (Gao et al., 2019), catheter-based radiofrequency ablation (Krum et al., 2009), endovascular ultrasound (Fengler et al., 2019), injection of neurotoxic agents (Lohmeier and Hall, 2019), external stereotactic radiofrequency (Cai et al., 2019), external high-intensity focused ultrasound (Wang et al., 2013), etc.,] might reduce BP through sympathetic nerve activity inhibition. However, these procedures of current neuromodulation methods are either invasive or associated with complete nerve damage. The invasive surgery for the implant of FES stimulator would lead to some difficulties and/or adverse effect such as complicated surgical implantation and perioperative and post-surgery risks. In addition, dealing with the damaged implanted electrodes wrapped in the scar tissue remains unclear and difficult (Plachta et al., 2014). Besides that, despite that the catheter-based RSD procedures are minimally invasive and the procedures of performing external stereotactic radiofrequency or external high-intensity focused ultrasound for RSD are non-invasive, BP is reduced by completely destroying the renal sympathetic nerve by utilizing the high intensity of the radiofrequency/ultrasound energy.

By contrast with the electrical approaches (such as FES) and denervation methods (such as RSD), one emerging technology that is called low-intensity focused ultrasound stimulation (FUS) has been shown in a number of literatures to be promising in non-invasive neuromodulation without damaging the nerve (Baek et al., 2017; Landhuis, 2017); thus, it should be a potential approach for BP modulation. Specifically, the penetrability of ultrasound allows it to penetrate non-invasively from the body surface into a deep targeted nerve or tissue without a need of surgical implantation. The focused characteristics of ultrasound could ensure the precise stimulation of a targeted nerve or tissue. Furthermore, by setting different acoustic parameters such as intensity and frequency, neural activity could be selectively activated or inhibited without nerve damage. Owing to those advantages, FUS has opened a new era for non-invasive neuromodulation and has been recently applied in a number of studies in the field of neurosciences (Hakimova et al., 2015; Baek et al., 2017; Landhuis, 2017). By far, the FUS neuromodulation technique has been widely applied for brain stimulation (Hakimova et al., 2015; Baek et al., 2017; Landhuis, 2017). Furthermore, the FUS has also been used to target different peripheral nerves for neuromodulations. For example, targeting FUS at the retina could activate a visual-evoked potential equal to strong visual responses (Menz et al., 2013), at ear labyrinth it could cause auditory sensation corresponding to an audio-modulating signal (Tsirulnikov et al., 1988; Gavrilov and Tsirulnikov, 2012), at the peripheral sensory neuroreceptors or nerve fibers it could excite tactile, thermal, and pain sensations (Bystritsky et al., 2011; Gavrilov and Tsirulnikov, 2012; Legon et al., 2012), and at the sciatic nerve it could modulate motor neuron activity (Kim et al., 2020).

The successful and the promising applications of low-intensity FUS as described above for the neuromodulation of both the central and the peripheral nerves inspired us to consider the feasibility of utilizing FUS technique to stimulate the peripheral nerves (such as vagus nerve) for BP regulation. One previous animal study suggested that using focused pulsed ultrasound for vagus nerve modulation could induce the change of its compound action potential (CAP) (Juan et al., 2014). As it is well known, the BP value is regulated by the vagus nerve (Plachta et al., 2014; Annoni et al., 2019). Hence, we hypothesized that it might be feasible to control the BP by stimulating the vagus nerve *via* low-intensity FUS. In this pilot study, by using animals, we investigated whether BP could be effectively controlled through low-intensity FUS neuromodulation and explored how the different acoustic intensities would influence on the BP modulation as well as heart rate (HR). This study would be worth looking forward to provide an effective way for non-invasive and non-drug management of hypertension.

## Materials and Methods

**FIGURE 1:**
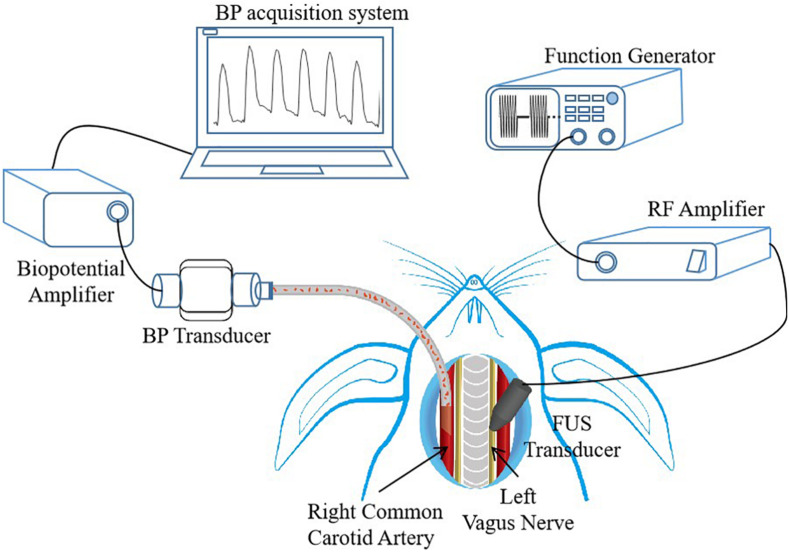
Experimental setup, with description of animal preparation, focused ultrasound stimulation (FUS) control, and data acquisition system. The placements of FUS transducer and blood pressure (BP) transducer are shown. RF, radio frequency.

In this study, the experiments of BP control through FUS modulation were conducted on eight white rabbits (six New Zealand white rabbits and two Japanese white rabbits, all male, body weight 3.5–4.5 kg). For each rabbit, the BP modulation experiments included three sections: (1) animal preparation, (2) ultrasonic stimulation, and (3) BP data acquisition (as shown in Figure 1). All the animal experimental procedures were approved by the Institutional Animal Care and Use Committee (IACUC) of Shenzhen Institutes of Advanced Technology, Chinese Academy of Sciences (SIAT-IACUC-190801-YGS-LWH-A0454-01). The details of the animal experiment are described in the following subsections.

### Animal Preparation

For each rabbit, its left vagus nerve was chosen as the targeted nerve of BP modulation, and its right common carotid artery was selected as BP detecting site. Although the FUS can be applied non-invasively, a surgery was performed to expose the left vagus nerve to ensure that the focus of the ultrasound transducer precisely stimulates the targeted vagus nerve, and the right common carotid artery was also exposed by surgery to measure the BP values. Before the surgery commenced, a face mask was initially put on the face of the rabbits. The rabbits were induced to anesthetize using 5% isoflurane delivered with oxygen at a rate of 0.8 L/min, and then the anesthesia level was reduced to 2.5% isoflurane for maintenance. After that, the rabbits were placed on a platform in supine position, their neck hairs were shaved off with a razor, and then the surgical area was sterilized with alcohol. The underlying sternohyoid muscle was exposed through a ventral neck incision, and then the left vagus nerve and the right common carotid artery were exposed and separated from the neurovascular bundles, respectively. The exposed left vagus nerve was targeted with a low-intensity FUS probe for neuromodulation, and the exposed right common carotid artery was catheterized for continuous BP wave recording. During the experiment, 0.3% heparin sodium, an anticoagulant, was used to prevent blood coagulation when necessary to ensure that the experiment goes on smoothly. After successfully conducting the experiment, the rabbits were sacrificed with an overdose of isoflurane.

### Ultrasonic Stimulation

#### Sonication Setup and Acoustic Measurement

An ultrasonic stimulation system was built using a function generator, a power amplifier, and a focused ultrasound transducer (shown in Figure 1). The driving signal from the functional generator (SDG 1032X, SIGLENT, Shenzhen, China) was amplified by a power amplifier (A075, E&I, Ltd., Rochester, NY, United States) and then sent to a focused ultrasound transducer. The focused ultrasound transducer with a fundamental frequency (FF) of 3.7 MHz, a diameter of 19.5 mm, and a focal length of 17 mm was connected to an acoustic collimator. The collimator was designed based on the characteristics of the ultrasound transducer and was fabricated with a three-dimensional (3D) printer, which was used to precisely focus the ultrasound on the stimulation target. During the experiment, the collimator was filled with ultrasound gel for better acoustic coupling.

**FIGURE 2:**
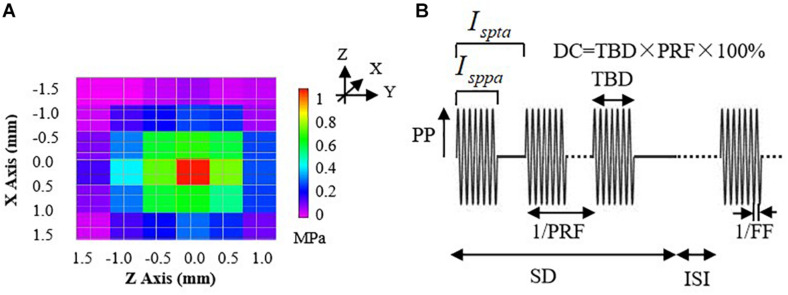
**(A)** The acoustic pressure distributions of the 3.7-MHz FUS transducer in the axial plane (X–Z section) with 0.5-mm steps at the focus position (*Y*-axis). **(B)** An illustration of parameters for a typical pulsed sonication: PP, peak pressure; *I*_sppa_, spatial-peak pulse-average intensity; *I*_spta_, spatial-peak time-average intensity; DC, duty cycle; TBD, tone-burst duration; PRF, pulse repetition frequency; SD, sonication duration; ISI, inter-stimulus interval; FF, fundamental frequency.

Using the FUS with different acoustic intensities to stimulate a nerve may cause different biological responses. In this study, two kinds of acoustic intensities, spatial-peak pulse-average intensity (*I*_*sppa*_) and spatial-peak time-average intensity (*I*_spta_), were examined to explore their influences on BP modulation. The *I*_*sppa*_ and the *I*_spta_ represent the degree of acoustic pressure given by the driving voltage and the energy deposition rate in the tissue, respectively. The *I*_*sppa*_ can be theoretically calculated according to the American Institute of Ultrasound Medicine standards (NEMA, 2004) using the following equation (1):

(1) $$I_{\text{sppa}}=\frac{P_{0}^{2}}{2\,\rho \,c}$$

where *P*_0_ is the acoustic peak pressure, ρ is the density of the medium (1,000 kg/m^3^), and *c* is the sound speed in the medium (1,480 m/s). Before the experiments, three sets of *I*_*sppa*_ parameters (shown in Table 1) were determined, and the acoustic pressure fields in the focal region generated by the ultrasonic stimulation system were practically measured using a 3D acoustic scanning system (UMS3, Precision Acoustics, Dorchester, United Kingdom) equipped with a calibrated needle-type hydrophone (HNP-0400, Onda, Sunnyvale, CA, United States). Figure 2A shows a typical example of acoustic pressure distributions in the axial plane (X–Z section) with a 0.5-mm step at the focus position (*Y*-axis).

**TABLE 1:** Sonication parameters used in the stimulation trials with fixed fundamental frequency (3.7 MHz), sonication duration (5 s), and inter-stimulus interval (1 s).

AI: *I*_*sppa*_ (W/cm^2^)	Duty cycle (%)	Tone-burst duration (ms)	AI: *I*_spta_ (W/cm^2^)
18.0	30	0.3	5.4
48.5	30	0.3	14.6
87.3	30	0.3	26.2
18.0	50	0.5	9.0
48.5	50	0.5	24.3
87.3	50	0.5	43.6
18.0	70	0.7	12.6
48.5	70	0.7	34.0
87.3	70	0.7	61.1
18.0	100	–	18.0
48.5	100	–	48.5
87.3	100	–	87.3

The *I*_spta_ can be calculated using the equation (2):

(2) $$I_{\text{spta}}=\text{DC}\times I_{\text{sppa}}$$

where DC represents the ultrasound duty cycle, a percentage ratio of sonication active time to a total period. Thus, the *I*_spta_ would be determined by both acoustic peak pressure and time-related parameters (DC). During the experiment, the *I*_spta_ values ranged from 5.40 to 87.3 W/cm^2^ by setting the DC values to be 30, 50, 70, and 100%, with different *I*_*sppa*_ values (18.0, 48.5, and 87.3 W/cm^2^), as shown in Table 1.

#### Ultrasound Stimulation Trials

The FUS trials were conducted by setting parameters of the function generator to examine the effects of different acoustic intensities on the BP regulation. One channel of the function generator was used to control the ultrasound FF and tone-burst duration (TBD) and was triggered by another channel of the function generator which was used to generate the bursts of sinusoidal pulse waves and control the pulse repetition frequency (PRF), sonication duration (SD), and inter-stimulus interval (ISI), as shown in Figure 2B. The duty cycle (DC) equals to TBD divided by 1/PRF. Note that the FUS with a DC of 100% represents continuous stimulation and that with a DC less than 100% represents the pulsed stimulation.

In this study, the FF, PRF, SD, and ISI were fixed and set to 3.7 MHz, 1 kHz, 5 s, and 1 s, respectively, and the TBD was changed with different values to obtain different DC and *I*_spta_, as listed in Table 1. For each trial with a set of predefined sonication parameters, the FUS duration lasted for about 20 s to clearly observe the BP changes, and then we waited for around 60 s after cessation of the stimulation until the BP returned to the baseline level. The sequence of sonication trials was pseudo-randomized and balanced across the animals. Repeated ultrasound stimulation trials on the same animal were conducted to ensure the effectiveness of the different sonication parameter sets.

### Data Acquisition

During the FUS experiments, the BP waveform was continuously recorded in the exposed right common carotid artery with a commercially available data acquisition system (ADInstruments Pty Ltd., Bella Vista, NSW, Australia). The sampling rate was set as 1,000 Hz. Four important cardiovascular parameters, beat-to-beat systolic BP (SBP), diastolic BP (DBP), mean arterial pressure (MAP), as well as heart rate (HR), were calculated from the BP recordings by using a commercial software (LabChart toolbox, ADInstruments). SBP and DBP are defined as the amplitude of the peak and the trough of BP waveform, respectively. MAP represents an average blood pressure within a single cardiac cycle, which could be calculated using equation (3). HR is defined as the number of heartbeats in a minute and herein is calculated by equation (4).

(3) $$\text{MAP}=\frac{1}{3}\,\text{SBP}+\frac{2}{3}\,\text{DBP}$$

(4) HR=60/IBI

where IBI indicates inter-beat interval.

### Statistical Analysis

A paired *t*-test was conducted to assess the significant difference of the changes in arterial BP and HR when the FUS was turned off and on. A correlation analysis measured with Pearson coefficient was performed to estimate the relationship between the BP control and each of the ultrasonic stimulation parameters, as well as the relevance between the BP changes and the HR changes in response to the FUS. The analysis results were expressed as mean ± standard deviation (SD). A value of *p* < 0.05 was considered to be statistically significant. All statistical tests were conducted using the SPSS software package for data analysis.

## Results

### BP Modulations With a Low-Intensity FUS to the Vagus Nerve

**FIGURE 3:**
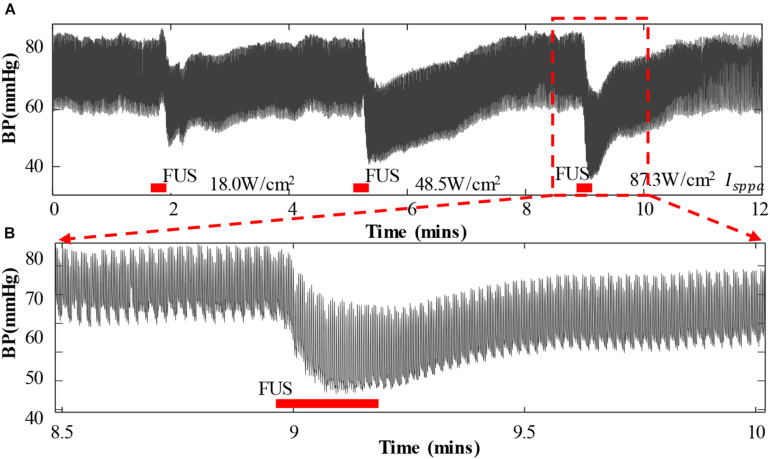
Arterial blood pressure (ABP) and heart rate change when applying focused ultrasound stimulation (FUS) to the vagus nerve. **(A)** A typical recording of the original ABP waveform of a white rabbit responding to the FUS with increasing acoustic intensities (18.0, 48.5, and 87.3 W/cm^2^ *I*_*sppa*_). ABP decreased when the FUS was turned on. **(B)** A zoom-in view of ABP waveform under the FUS at 87.3 W/cm^2^ *I*_*sppa*_. The red bar indicates the period of stimulation.

Figure 3A shows a representative segment of the continuous BP waveform recordings during the FUS to the left vagus nerve of a rabbit with an incremental acoustic intensity (18.0, 48.5, and 87.3 W/cm^2^ *I*_*sppa*_), in which the FUS durations were indicated by red bars. It was clearly observed from Figure 3A that the arterial BP waveform gradually decreased from the baseline level when the FUS was turned on, and the BP waveform slowly returned back to the baseline level when the FUS was turned off. Similar changes in the BP waveform characteristics were observed when the FUS was conducted repeatedly. With the increase of acoustic intensity (from 18.0 to 87.3 W/cm^2^ *I*_*sppa*_), the BP value decreased more substantially. To further illustrate this phenomenon in a clearer manner, a zoom-in of the BP waveform under the FUS at 87.3 W/cm^2^ *I*_*sppa*_ is shown in Figure 3B.

**FIGURE 4:**
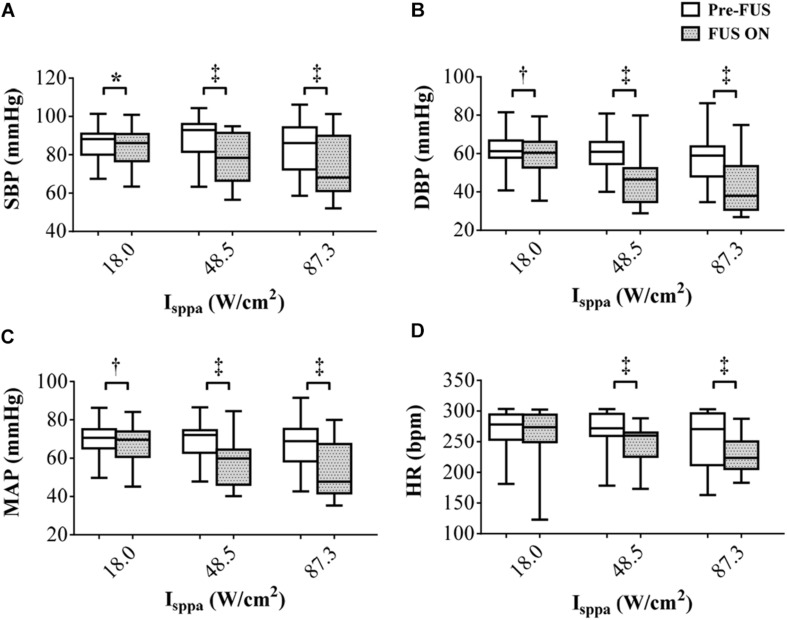
The overall changes (*n* = 8) in **(A)** systolic blood pressure, **(B)** diastolic blood pressure, **(C)** mean arterial pressure, and **(D)** heart rate responding to focused ultrasound stimulation. The white boxes indicate the values before stimulation, and the gray boxes indicate the values during the stimulation. *p < 0.05, †*p* < 0.01, ‡*p* < 0.001.

Figure 4 shows the overall changes of SBP, DBP, MAP, and HR of eight rabbits when applying FUS on the left vagus nerve at 3.7 MHz FF, 1 kHz PRF, 5 s SD, and 18.0–87.3 W/cm^2^ *I*_*sppa*_. The white boxes indicated the values before stimulation, and the gray boxes demonstrated the values recorded during the stimulation. As shown in the boxplots, the mean values of SBP decreased by 1.91, 9.71, and 10.09 mmHg when applying FUS at 18.0, 48.5, and 87.3 W/cm^2^ *I*_*sppa*_, respectively. Meanwhile, the mean values of DBP decreased by 3.01, 13.75, and 14.55 mmHg, MAP decreased by 2.64, 12.41, and 13.06 mmHg, and HR decreased by 7.02, 19.21, and 24.90 bpm when applying FUS at 18.0, 48.5, and 87.3 W/cm^2^ *I*_*sppa*_, respectively. A paired *t* test showed that the decrease in SBP, DBP, and MAP was significant when applying FUS at 18.0, 48.5, and 87.3 W/cm^2^ *I*_*sppa*_ (Figures 4A–C). HR was significantly decreased at 48.5 and 87.3 W/cm^2^ *I*_*sppa*_ (Figure 4D). Thus, FUS on the left vagus nerve at 3.7 MHz FF, 1 kHz PRF, 5 s SD, 1 s ISI, and 18.0–87.3 W/cm^2^ *I*_*sppa*_ significantly decreased SBP, DBP, and MAP. The effectiveness and the repeatability of the SBP, DBP, MAP, and HR reductions in response to the FUS on the left vagus nerve were validated by multiple stimulation trials under different stimulation parameters.

### Effect of Acoustic Intensity on BP Modulations

#### *I*_spta_

**FIGURE 5:**
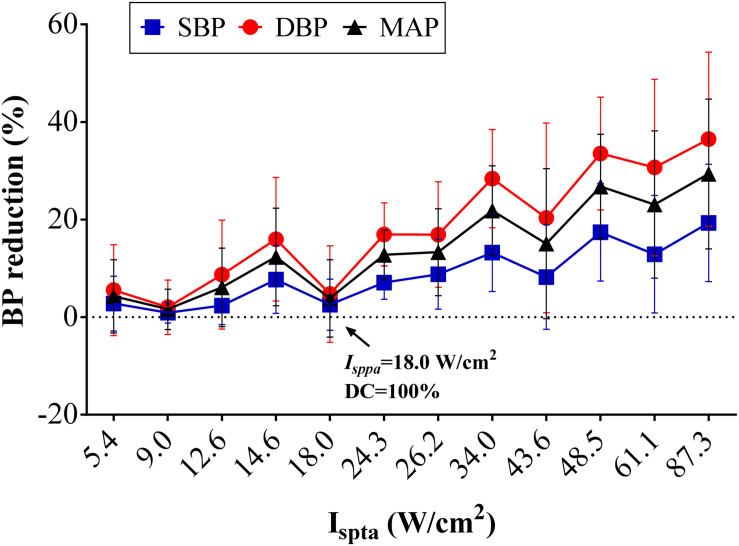
Effects of acoustic intensity (AI) on blood pressure (BP). The overall BP changes (*n* = 8) in percentage relative to the baseline level in the different AIs (*I*_spta_) with the set 3.7-MHz fundamental frequency, 1-kHz pulse repetition frequency, and 5-s sonication duration. Reductions in systolic blood pressure, diastolic blood pressure, and mean arterial pressure increased with AI.

Figure 5 shows the reduction percentage of SBP, DBP, and MAP as *I*_spta_ increases when applying FUS on the left vagus nerve at 3.7 MHz FF, 1 kHz PRF, and 5 s SD. On average, decreases of 0.92–19.33% in SBP, 2.04–36.49% in DBP, and 1.61–29.36% in MAP were recorded when *I*_spta_ increased from 5.40 to 87.30 W/cm^2^. Furthermore, the decrease in SBP, DBP, and MAP was significantly correlated with *I*_spta_ (*r* = 0.55, *p* < 0.01 for SBP reduction; *r* = 0.62, *p* < 0.01 for DBP reduction; *r* = 0.61, *p* < 0.01 for MAP reduction). Hence, the results suggested that the BP modulations had a significant correlation with acoustic intensity (*I*_spta_) when applying FUS on the left vagus nerve. The SBP, DBP, and MAP reduction tends to increase more substantially with a higher *I*_spta_. However, it is also worthy to note that the SBP, DBP, and MAP reductions are not completely monotonically increasing as *I*_spta_ increases. Note that *I*_spta_ is composed of stimuli with different DC and *I*_*sppa*_ combinations, and the DC and *I*_*sppa*_ might contribute to the BP reduction differently, which might explain the non-monotonicity between *I*_spta_ increase and BP reduction. Therefore, the respective effects of *I*_*sppa*_ and DC on BP modulations are presented in the following discussion.

#### *I*_*sppa*_ and DC

**FIGURE 6:**
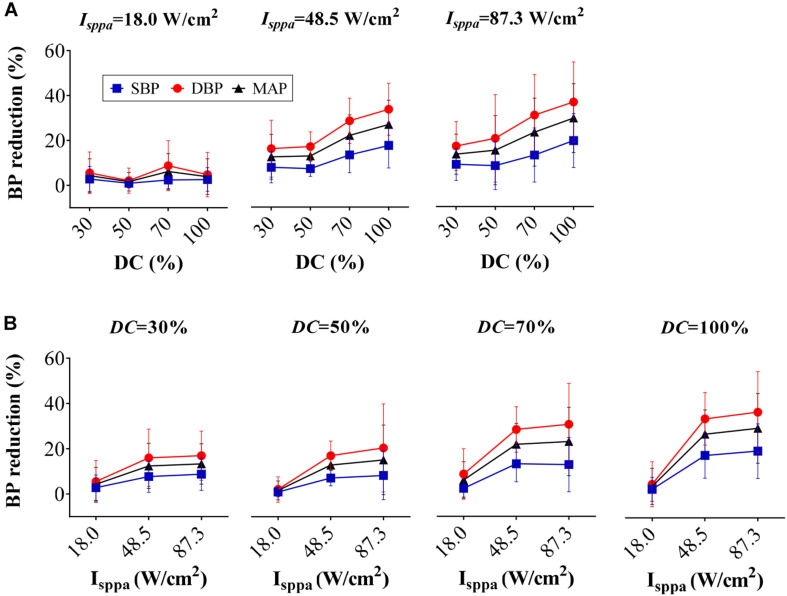
Reduction percentage of systolic blood pressure, diastolic blood pressure, and mean arterial pressure as **(A)** duty cycle increases and **(B)** *I*_*sppa*_ increases.

Figure 6A shows the reduction percentage of SBP, DBP, and MAP as DC increases when applying FUS with *I*_*sppa*_ fixed at 18.0, 48.5, and 87.3 W/cm^2^, respectively. As shown in the figure, BP reduction increased monotonically with the rise of DC when the *I*_*sppa*_ was fixed. A correlation analysis further confirmed that the magnitude of SBP, DBP, and MAP reduction was also significantly correlated with DC (*r* = 0.29, *p* < 0.05 for SBP reduction; *r* = 0.31, *p* < 0.01 for DBP reduction; *r* = 0.31, *p* < 0.01 for MAP reduction). Figure 6B shows the reduction percentage of SBP, DBP, and MAP as *I*_*sppa*_ increases when applying FUS with DC fixed at 30, 50, 70, and 100%, respectively. In general, when the DC was fixed, the SBP, DBP, and MAP reductions increase monotonically with the rise of *I*_*sppa*_. Furthermore, the magnitude of SBP, DBP, and MAP reduction was also significantly correlated with *I*_*sppa*_ (*r* = 0.42, *p* < 0.01 for SBP reduction; *r* = 0.49, *p* < 0.01 for DBP reduction; *r* = 0.48, *p* < 0.01 for MAP reduction). Thus, both *I*_*sppa*_ and DC could affect the magnitude of BP reduction, which might explain the non-monotonic relationship between *I*_spta_ and BP reduction (as shown in Figure 5). As labeled in Figure 5, FUS with *I*_spta_ of 18.0 W/cm^2^ produced a relatively lower BP induction than the adjacent points; this might be because of the small *I*_*sppa*_ (18.0 W/cm^2^), leading to a smaller BP decline.

#### Effect of HR Changes on BP Modulations

**FIGURE 7:**
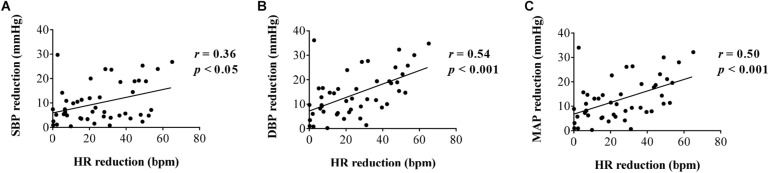
Correlation analysis between heart rate reduction and **(A)** systolic blood pressure reduction, **(B)** diastolic blood pressure reduction, and **(C)** mean arterial pressure reduction, respectively.

In order to investigate the effect of HR changes on BP modulation, a correlation analysis between the changes of HR and changes of BP was conducted, and the results are shown in Figure 7. As can be seen from the figure, the decrease in SBP, DBP, and MAP was significantly correlated with the decrease in HR (*r* = 0.36, *p* < 0.05 for SBP reduction; *r* = 0.54, *p* < 0.001 for DBP reduction; *r* = 0.50, *p* < 0.001 for MAP reduction). It demonstrates that the HR changes have an important effect on BP regulation. However, the correlation coefficients are moderate (0.36–0.54, the maximum scope is [0 1]), which demonstrates that the changes of BP elicited by FUS are not completely caused by HR changes. There are other factors that might influence the changes in BP.

## Discussion and Conclusion

Clinically, the first-line treatment for patients with hypertension would be pharmacological antihypertensive therapy. Currently, while there are a plethora of available antihypertensive drugs that have been used to effectively treat hypertension, the drug safety issue has been sustainedly concerned by the physicians and the patients (Husserl and Franz, 1981; Psaty et al., 1995; Klungel et al., 2001; Marc et al., 2019). In addition, the need of administrating BP is unmet in the resistant hypertensive population. Therefore, developing devices-based neuromodulation approaches would be one alternative way to treat hypertension and its comorbidities (Scheffers et al., 2010; Bisognano et al., 2011; Lohmeier and Iliescu, 2011; Bakris et al., 2012; Hoppe et al., 2012). Recently, a new advanced neuromodulation technology that uses focused ultrasound stimulation to suppress or boost neurons’ activity has been approved to be promising to treat some neuropathies such as movement disorders, depression, and anxiety (Bystritsky et al., 2011; Legon et al., 2012; Lohmeier and Hall, 2019). Whether the newly approved neuromodulation technique would be feasible and effective for BP modulation was investigated in this animal study.

In this pilot study, the acute response of BP under low-intensity FUS to the vagus nerve of rabbits was investigated. The experimental results indicated that BP could be effectively modulated through low-intensity FUS technique when appropriate sonication parameters were set, to the best of our knowledge, which should be the first time to demonstrate the feasibility of using FUS on peripheral nerve for BP neuromodulation. When FUS was targeted at the vagus nerve with the sonication parameters of 3.7 MHz FF and 18.0–87.3 W/cm^2^ *I*_*sppa*_, the values of the SBP, MAP, and DBP were observed to be significantly reduced. Meanwhile, the acoustic intensities of the FUS had a significant effect on the degree of BP variation, such that the higher the acoustic intensity, the more substantial reduction in the values of the SBP, DBP, and MAP. In addition, the HR also decreased during the FUS period, which indicates that the FUS at the vagus nerve may modulate BP through regulating cardiac function and peripheral vascular function. The correlation analysis between BP and HR produced intermediate values (*r* = 0.36 for ΔSBP, *r* = 0.54 for ΔDBP, *r* = 0.50 for ΔMAP), demonstrating that the reduction of BP was not only induced by the decline of HR. The decrease of HR when the FUS was utilized also suggested that low-frequency FUS of vagus nerve plays a role on the relief of tachycardia but should avoid bradycardia.

It is worth noting that, compared to the FES-based neuromodulation approach that has been proposed for hypertension managements in clinical trials (Hoppe et al., 2012), the acute response of BP to the proposed FUS approach was found to be similar as that of the FES reported in a previous study (Plachta et al., 2014). It was also found that the values of the SBP, DBP, and MAP reduced substantially when the focused ultrasound stimulation was turned on and then returned back to the baseline level when the stimulation was turned off. BP response exhibited similar waveform characteristics when a repeated stimulation was administered during the experiment (as illustrated in Figure 3). This suggested that the FUS approach could be promising as an alternative non-drug treatment method for hypertension, similar to the FES. In comparison with applying the FES for BP regulation, which requires the implantation of the stimulator into the targeted nerve by invasive surgery (Scheffers et al., 2010; Bisognano et al., 2011; Lohmeier and Iliescu, 2011; Bakris et al., 2012; Hoppe et al., 2012; Plachta et al., 2014; Gierthmuehlen et al., 2016; Annoni et al., 2019), ultrasound energy could penetrate into the deep tissue in a non-invasive way, which has been proven in a lot of previous studies (Bystritsky et al., 2011; Gavrilov and Tsirulnikov, 2012; Baek et al., 2017), that may make the proposed FUS approach outperform the existing device-based methods for BP regulation. In addition, unlike the high-intensity focused ultrasound stimulation used to ablate the renal sympathetic nerve for drug-resistant hypertension treatment (Wang et al., 2013), FUS could induce an antihypertensive effect without damaging the nerve or tissues surrounding it. Therefore, FUS might provide a better way for non-invasive and non-drug management of hypertension. Although the FUS can be applied non-invasively, which has been proven to be feasible in lots of previous studies (Baek et al., 2017; Landhuis, 2017), in this pilot study, it is noteworthy that, in order to identify the exact targeted nerve that could induce an obvious antihypertensive effect through FUS, an invasive animal surgery model was used to expose the target nerve, and the ultrasound energy was directly focused on the target nerve using a collimator fully filled with a coupling agent. After identifying the target nerves specifically, future works would test the effect of BP control by non-invasive stimulation from the body surface. Whereas delivering the FUS with exact alignment to the target nerve is a challenging issue, image-guided technique may provide a promising option to address the alignment issue, which has been proven to be feasible in some previous studies (Kim et al., 2020).

**FIGURE 8:**
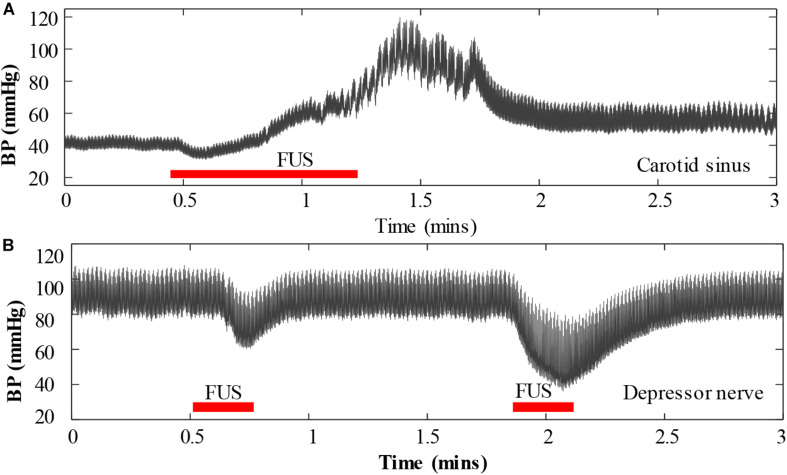
An example of the response of blood pressure when applying focused ultrasound stimulation to a carotid sinus nerve **(A)** and to a depressor nerve **(B)**.

The vagus nerve was initially selected in this pilot study because BP is more responsive to changes in vagus nerve activity than those in other nerves, and the vagus nerve is relatively easy to locate. However, other autonomic nerves such as sympathetic nerve and stretch-sensitive nerves such as carotid sinus nerve and depressor nerve may also be used as a targeted nerve for BP modulation with FUS method. In this study, we also preliminarily investigated the response of BP when applying FUS to the carotid sinus nerve and to the depressor nerve, respectively. However, the experimental results showed that the sonication parameters used in this study increased BP rather than decreased it when FUS was targeted at the carotid sinus nerve, and when FUS was targeted at the depressor nerve, it could reduce BP similar to that of targeting the vagus nerve, but the BP decrease induced by the depressor nerve stimulation was not as significant as that induced by the vagus nerve stimulation. This phenomenon was also similar as electrical stimulation (Douglas and Ritchie, 1956). An example of the response of BP when applying FUS to a carotid sinus and to a depressor nerve is shown in Figures 8A,B, respectively. The intensity (*I*_spta_) of the FUS used in Figure 8A was 34 W/cm^2^ and in Figure 8B were 14.6 and 26.2 W/cm^2^, respectively. Future works will explore the appropriate sonication parameters that could induce an antihypertensive effect when FUS was targeted to the carotid sinus nerve, which may further broaden the window of understanding on the potential applications of FUS for BP regulations in clinical practice.

Noting that FF of ultrasound is an important parameter for FUS, according to previous literatures (Bystritsky et al., 2011; Gavrilov and Tsirulnikov, 2012; Baek et al., 2017), various FF parameters have been adopted for FUS neuromodulation. Generally, low-frequency ultrasound with FFs of less than 1 MHz was mostly utilized, while some studies adopted relatively high FFs such as 1.68, 1.9, 2.5, 2.7, 3.2, 3.5, 4.6, 5, 2–7, and 8 MHz for FUS neuromodulation. In order to learn the response of BP induced by FUS at different frequencies, three ultrasonic transducers with FF values of 548 kHz, 1.05 MHz, and 3.7 MHz were tested in this preliminary experiment, respectively. Our results showed that the 548-kHz ultrasonic transducer induced little BP response, the 1.05-MHz ultrasonic transducer induced a slight BP response, and the 3.7-MHz ultrasonic transducer induced a significant BP response. Thus, a 3.7-MHz ultrasonic transducer was used in this pilot study, and its effect on BP modulation was systematically investigated. Other ultrasonic parameters, such as PRF, SD, and ISI, were also chosen based on previous literatures (Bystritsky et al., 2011; Gavrilov and Tsirulnikov, 2012; Baek et al., 2017) and preliminary experimental results.

In summary, for the first time, to the best of our knowledge, this pilot animal study provided the evidence of acute response of BP by low-intensity FUS. The experimental data indicated that BP could be effectively modulated through low-intensity FUS of the vagus nerve when the sonication parameters were appropriately determined. The acute response of BP to low-intensity FUS was similar as that of electrical stimulation, which indicates that this new approach may provide an alternative way for non-invasive and non-drug management of hypertension and other diseases associated with vagal activity modulation. However, the long-term antihypertensive effect still needs to be verified by chronic stimulation. In the future, the influence of additional sonication parameters such as FF, PRF, SD, and ISI on short-term and long-term BP attenuation/regulation as well as its corresponding thermal effect would be further investigated. In addition, the mechanism by which FUS induces BP response remains to be further investigated.

## Data Availability Statement

The original contributions presented in the study are included in the article/supplementary material, further inquiries can be directed to the corresponding author.

## Ethics Statement

The animal study was reviewed and approved by the Institutional Animal Care and Use Committee (IACUC) of Shenzhen Institutes of Advanced Technology, Chinese Academy of Sciences.

## Author Contributions

GL initiated and supported this project and checked through all the writing. FC and LX participated in the design of the study and provided technical supports. W-HL and NJ carried out the clinical examination, participated in the work of analysis, and drafted the manuscript. JH carried out the clinical examination and provided clinical support. All the authors read and approved the final manuscript.

## Conflict of Interest

The authors declare that the research was conducted in the absence of any commercial or financial relationships that could be construed as a potential conflict of interest.
